# *Plasmodium falciparum* resistance to artemisinin-based combination therapies: A sword of Damocles in the path toward malaria elimination

**DOI:** 10.1051/parasite/2018021

**Published:** 2018-04-20

**Authors:** Manel Ouji, Jean-Michel Augereau, Lucie Paloque, Françoise Benoit-Vical

**Affiliations:** 1 LCC (Laboratoire de Chimie de Coordination du CNRS), BP 44099, 205 Route de Narbonne, 31077 Toulouse cedex 4 France; 2 Université de Toulouse; UPS, INPT; LCC; F-31077 Toulouse France

**Keywords:** malaria, artemisinin, drug resistance, parasite, *Plasmodium falciparum*

## Abstract

The use of artemisinin-based combination therapies (ACTs), which combine an artemisinin derivative with a partner drug, in the treatment of uncomplicated malaria has largely been responsible for the significant reduction in malaria-related mortality in tropical and subtropical regions. ACTs have also played a significant role in the 18% decline in the incidence of malaria cases from 2010 to 2016. However, this progress is seriously threatened by the reduced clinical efficacy of artemisinins, which is characterised by delayed parasitic clearance and a high rate of recrudescence, as reported in 2008 in Western Cambodia. Resistance to artemisinins has already spread to several countries in Southeast Asia. Furthermore, resistance to partner drugs has been shown in some instances to be facilitated by pre-existing decreased susceptibility to the artemisinin component of the ACT. A major concern is not only the spread of these multidrug-resistant parasites to the rest of Asia but also their possible appearance in Sub-Saharan Africa, the continent most affected by malaria, as has been the case in the past with parasite resistance to other antimalarial treatments. It is therefore essential to understand the acquisition of resistance to artemisinins by *Plasmodium falciparum* to adapt malaria treatment policies and to propose new therapeutic solutions.

## Abstract (fr)

L’utilisation, dans le traitement du paludisme simple, de combinaisons thérapeutiques associant un dérivé de l’artémisinine et une molécule partenaire a largement contribué à une réduction significative de la mortalité due à cette pathologie dans les régions tropicales et subtropicales ainsi qu’une diminution de 18% de nombre de cas de 2010 à 2016. Cependant, ces progrès sont sérieusement menacés par la diminution de l’efficacité clinique des artémisinines caractérisées par des clairances parasitaires retardées et un taux de recrudescence élevé, signalés en 2008 à l’ouest du Cambodge. La résistance aux artémisinines s’est déjà étendue à plusieurs pays d’Asie du Sud-Est. De plus, il a été montré que la résistance aux molécules partenaires des artémisinines dans ces combinaisons thérapeutiques (ACT) a été facilitée suite à une diminution de la sensibilité à l’artémisinine. L’une des principales préoccupations est non seulement la propagation de ces parasites multi-résistants dans le reste de l’Asie, mais aussi leur apparition possible en Afrique subsaharienne, continent le plus touché par le paludisme, comme cela a été le cas dans le passé avec la résistance de parasites à d’autres traitements antipaludiques. Il est donc essentiel de comprendre l’acquisition de la résistance de *Plasmodium falciparum* aux artémisinines afin d’adapter les politiques de santé face au paludisme et de proposer de nouvelles solutions thérapeutiques.

**Main mechanisms of *Plasmodium falciparum* resistance**

Two main mechanisms of resistance drive *Plasmodium* resistance to antimalarial drugs. The first one is an efflux of the drug away from its action site due to mutations in different transporter genes (like *pfcrt* in chloroquine resistance) or an increased number of the gene copies (like *pfmdr1* copy number in mefloquine resistance). The second is a change in the parasite target due to mutations in corresponding genes (like, at the cytosol level, *dhfr* and *dhps* in sulfadoxine-pyrimethamine resistance or, at the mitochondrion level, cytochrome b in atovaquone resistance). Surprisingly, resistance of falciparum malaria to the new artemisinin compounds involves a novel mechanism corresponding to a quiescence phenomenon.

## Introduction

Malaria is widespread in countries located in tropical and sub-tropical regions, where an estimated 3.2 billion people, nearly half of the world’s population, are at risk of infection [79]. Among the five species of *Plasmodium* that infect humans, *Plasmodium falciparum* is the most virulent, with the highest rates of complications and mortality as well as the most frequent incidence of red blood cell disorders worldwide [12]. Of the estimated 216 million cases in 2016, falciparum malaria accounted for 99% of cases in Africa, 77% of cases in the Western Pacific Region, 66% of cases in Southeast Asia, 58% of cases in the Eastern Mediterranean Region, and 36% of cases in America [79]. Over 91% of the estimated 445 000 global deaths from malaria in 2016 occurred in Sub-Saharan Africa, primarily among children less than five years of age [13,79]. Over the last 17 years, important measures have been put in place to prevent malaria, leading to a 60% reduction in its worldwide death toll. A decrease of 18% in the incidence of malaria cases was also reported from 2010 to 2016 [79]. This significant decrease in malaria incidence is the result of both preventive measures, such as the massive distribution of insecticide-treated nets, vector control strategies, and rapid diagnostic tests, as well as the use of artemisinin-based combination therapies (ACTs) in curative therapy. ACTs, recommended by the World Health Organization (WHO), are currently used as the first-line antimalarial treatment worldwide [79]. However, the current efforts to reduce the global burden of malaria are threatened by the rapid emergence and spread of *P. falciparum* resistance to ACTs including artemisinin derivatives and their partner drugs.

## Artemisinin and ACTs

The 2015 Nobel Prize in Medicine was awarded to Professor Tu Youyou for her key contribution to the discovery of artemisinin. Artemisinin, isolated from the plant *Artemisia annua*, and its semi-synthetic derivatives (artemether, artesunate, dihydroartemisinin) are powerful medicines known for their ability to swiftly reduce the number of *Plasmodium* parasites in the blood of patients suffering from malaria [2]. The unique characteristic of artemisinins is that they clear parasitemia more rapidly than all other antimalarials, including quinine [21]. Their efficacy can be ascribed to the fact that these compounds target not only the late erythrocytic parasite stages, like most antimalarial drugs, but also the early stages. Artemisinins, by killing the ring stage forms, allow the parasite to be pitted out of the host red blood cells, hence removing them from circulation [12,51,78] and preventing these parasite stages from maturing and sequestering in the vessels. This phenomenon is important in the pathogenesis because mature parasites are able to adhere to endothelial cells, blood cells and platelets which prevent their circulation in the bloodstream and they will therefore be able to escape retention by the spleen [12].

In the parasitic food vacuole, artemisinins react with haem that is generated from the digestion of haemoglobin and is toxic to the parasite, to form haem-artemisinin adducts [67]. These adducts seem to interact with *P. falciparum* haem detoxification proteins and inhibit haemozoin polymerisation leading to haem accumulation. Artemisinins are also responsible for alkylation of parasite proteins. Together, these events cause oxidative stress, leading to irreversible parasite damage and parasite death [17]. This explains the life-saving benefit of artemisinins and elucidates the mechanism underlying their superior efficacy for the treatment of malaria [21]. Artemisinins also reduce the number of gametocytes (sexual-stage parasites) responsible for its transmission to the vector, the *Anopheles* mosquito [2], both with direct anti-gametocyte activity and indirect action *via* the reduction of the asexual parasite population, which is the source of new gametocytes [18].

Since 1994, artemisinins have been used in ACTs to treat uncomplicated malaria. ACTs combine 2 active ingredients, artemisinins and another antimalarial drug, with different mechanisms of action. It has been reasoned that in ACTs, the partner drugs are chosen on the basis of their pharmacokinetic properties, which include much longer plasma half-lives (days to weeks) than those of artemisinins (1 to 2 h). While artemisinins are eliminated very rapidly from the body, the remaining parasites are exposed to the associated long-acting drug well after the end of the usual 3-day ACT course [80]. ACTs are the most effective antimalarial medicines available today, and they have replaced quinolines and antifolates as the first-line treatment for uncomplicated *P. falciparum* malaria in most endemic countries.

Five ACTs are currently used (Table 1), namely, artemether/lumefantrine (AL), artesunate/amodiaquine (ASAQ), artesunate/mefloquine (ASMQ), artesunate/sulfadoxine/pyrimethamine (AS+SP) and dihydroartemisinin/piperaquine (DHA/PPQ) [80]. A sixth ACT, artesunate/pyronaridine [47], was recently approved, and unfortunately, its current efficacy on day 42 was below 90% in Western Cambodia, an artemisinin-resistance area [38]. In 2016, 409 million ACT-based treatments were applied worldwide [79].

**Table 1:**
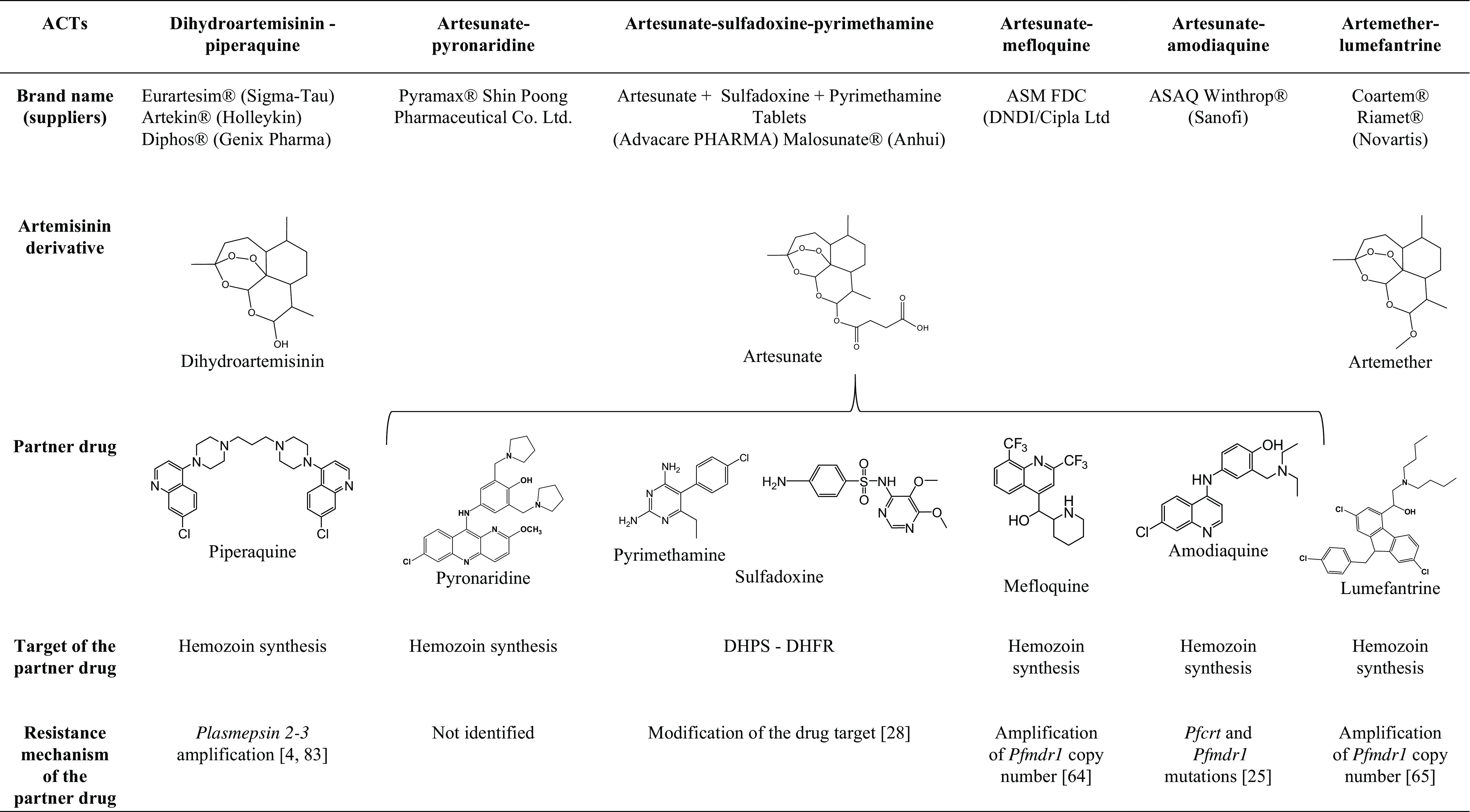
Artemisinin-based combination therapies.

DHPS = dihydropteroate synthase

DHFR = dihydrofolate reductase

Malaria treatment in pregnant women presents substantial risks for the mother and the unborn child. The risk-free use of artemisinin derivatives has not been well established in the first trimester of pregnancy [27], which is why the WHO does not recommend the use of these drugs during this period. Nevertheless, treatment with artemisinins during the second and third trimesters has been shown to be safe and does not have greater risks than other antimalarial drugs during these periods [32].

## Artemisinin resistance: definition

Artemisinin resistance was documented in 2008 on the Thailand-Cambodia border after artesunate monotherapy [22,53]. However, retrospective analysis indicates that artemisinin resistance likely emerged in 2001, before the widespread deployment of ACTs in Cambodia [81]. Artemisinin resistance was first correlated with delayed parasitic clearance after the first 3 days of treatment following artemisinin monotherapy or ACTs and higher rates of clinical failures due to increased parasitic recrudescence [22,53]. Increased clearance half-life was highly correlated with greater recrudescence rates *in vivo* after artemisinin elimination, which means that the parasites survive artemisinin treatment and are able to continue their development after the elimination of artemisinin from the body. Parasite clearance half-life is the time required to physiologically reduce the parasitemia by 50% following an administered antimalarial dose. Nevertheless, most patients who have delayed parasite clearance following treatment with an ACT are still able to clear their infection, as long as the partner drug remains effective. That is why delayed parasite clearance does not necessarily lead to treatment failure, even though artemisinin resistance can promote selection of the concomitant resistance to the partner drug.

Usually, in the resistance to other malaria drugs, the parasites are able to proliferate and multiply during treatment, but the same is not true in the case of artemisinin resistance. The resistance to artemisinin is based on a mechanism of entry into quiescence that occurs only at the ring stage. This finding was revealed by an experimental model, the F32-ART5 parasite line (a highly artemisinin-resistant strain established *in vitro* after 5 years of exposure to sequential and escalating concentrations of artemisinin reaching 7000-fold the IC_50_ value of the parental and sensitive F32-Tanzania strain) and was confirmed with Cambodian *P. falciparum* isolates [84,85]. As artemisinin resistance is due to the quiescent state of parasites in the presence of artemisinins [55], the standard *in vitro* chemosensitivity assays recommended by the WHO [19,22] to evaluate the antimalarial drug inhibition of parasite growth are not reliable tools for monitoring artemisinin resistance. The resistance to artemisinin is evidenced *ex vivo* or *in vitro* by the Ring stage Survival Assay (RSA^0-3h^), which is based on 6 h of 700nM dihydroartemisinin exposure in highly synchronised *P. falciparum* parasites at the ring stage and is followed by culture in drug-free conditions until the microscopic read-out at 72 h [84].

At a molecular level, a comparison of the whole genome sequence of F32-ART5 and of its twin sensitive parasite line, F32-TEM, demonstrated that a mutation in the propeller domain of the gene encoding Kelch protein 13 (K13) was associated with artemisinin resistance [6]. The exact function of this protein is not yet known, but it shares homologies with the human Keap1 protein, involved in the cell response to oxidative stress [6,1]. K13 is localised in the reticulum endoplasmic of the parasite [9].

**Figure 1:**
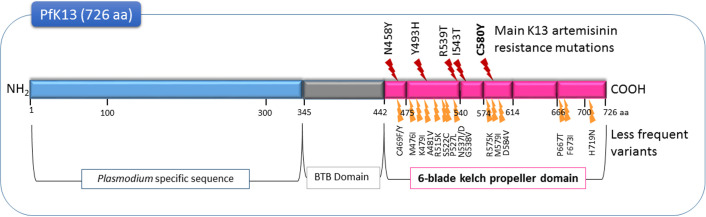
Mutations in the PfK13 protein involved in artemisinin resistance (WHO) [80].

Other non-synonymous mutations, all present after position 440 in the propeller domain of the *pfk13* gene (Figure 1), were also associated with artemisinin resistance in the field and were confirmed in laboratory experiments [80]. Only one of these mutations is sufficient to confer this resistance. However, not all reported non-synonymous propeller-domain K13 mutants indicate the emergence of artemisinin resistance [26]. That is why the relevance of a new *pfk13* mutation as a molecular marker of artemisinin resistance must be validated by clinical data and genetic engineering [79]. The high survival rate of parasites in RSA^0-3h^ correlates with both *pfk13* polymorphisms and clinical outcomes [6,74,80].

In summary, according to the WHO, artemisinin resistance is currently defined by delayed parasite clearance time at a clinical level, by a high survival rate of parasites in the Ring stage Survival Assay (RSA^0-3h^) *ex vivo* or *in vitro*, and by polymorphism of the *pfk13* gene [80].

## Current situation of *P. falciparum* resistance to artemisinins and to ACTs

At the present time, artemisinin resistance is limited to the Greater Mekong subregion, i.e., Cambodia, Thailand, Lao People’s Democratic Republic (Lao PDR), Myanmar, Viet Nam, and the Myanmar-China-India border area [77,80]. It has been demonstrated that artemisinin resistance has not only spread across this region but has also arisen independently several times in different locations [43,75]. ACTs were implemented to contain clinical resistance after artemisinin monotherapy, based on the different mechanistic and pharmacokinetic properties of the two combined antimalarials. Unfortunately, even though artemisinin resistance leads to few real clinical failures, it promotes selection for partner-drug resistance mainly due to mismatches in the pharmacokinetics of the two drugs, causing frequent treatment failure of ACTs [3]. An increase in concomitant artemisinin and partner drug resistances has been observed in recent years and, as a consequence, treatment failures after ACTs are becoming more widespread in Southeast Asia [24,39,50]. However, outside the Greater Mekong subregion, treatment failure with ACTs has occurred in the absence of artemisinin resistance mainly due to partner drug resistance [80].

In Southeast Asia, the first ACT was implemented in 1994 (artesunate-mefloquine (ASMQ) introduction in a refugee camp at the Thailand-Myanmar border [60]), before the WHO started recommending the use of ACTs as first-line antimalarial treatment in 2001. In 2006, the declining efficacy of ASMQ was suspected for the first time on the Cambodia-Thailand border [86]. Thereafter, ASMQ clinical failures were reported on the Thailand-Myanmar border in correlation with delayed parasite clearance time [50] and the amplification of *pfmdr1* gene copy numbers [48,58]. In addition, a prospective study conducted between 2003 and 2013 showed that the increasing prevalence of *pfk13* mutations was the decisive factor for the rapid decline in the efficacy of ASMQ on the Thailand-Myanmar border [60].

Clinical failures after dihydroartemisinin-piperaquine (DHA/PPQ) treatment have also been reported, first in Cambodia in 2013 [37] and later in Vietnam in 2017 [59,76], five and twelve years, respectively, after DHA/PPQ treatment introduction. DHA/PPQ resistance was confirmed by several reports and correlated with *pfk13* polymorphism, *plasmepsin 2-3* gene amplification and single copies of the *pfmdr1* gene [4,83]. Recent data suggest that piperaquine resistance has developed in a background of artemisinin resistance [3,29,60,68,71].

It is of concern that the emergence of artemisinin resistance in Southeast Asia involves severe malaria. Recently, two patients treated with artesunate, the treatment of choice for severe falciparum malaria, showed poor responses, and one patient died [63].

According to the latest WHO Malaria Report [79], an ACT is considered to present high risk of failure if high treatment failures for any partner drug in the corresponding ACTs are reported. Clinical failure rates greater than 10% have now been reported for the 5 ACTs in Cambodia, for 2 ACTs in Thailand and Lao PDR and for 1 ACT in Viet Nam, Myanmar, and in the Chinese and Indian border regions with Myanmar.

In such cases, the treatment must be replaced by another one, which will be evaluated every two years to adapt the treatment as quickly as possible [79]. A triple combination therapy regimen is one of the iterations of the Tracking Resistance to Artemisinin Collaboration project, known as TRAC II. This project is the first of its kind to investigate the safety, tolerability and efficacy of triple artemisinin-based combination therapies (TACTs) in clinical trials in Southeast Asia. In TACTs, the third partner drug should have an intermediate half-life so that it can provide an associative protective effect over both the fast-acting artemisinin drug and the long-acting partner drug [20,23,77].

## Is drug cycling an option for artemisinin resistance?

Susceptibility to chloroquine has been restored in Malawi and Zambia, and its re-introduction long after its withdrawal will show its efficacy in the field [33–35,49]. However, this recovered sensitivity is not systematic, as shown in Venezuela, where parasites remained resistant to chloroquine more than 15 years after the cessation of the use of chloroquine [16]. Thus, the use of drug “cycling”, based on alternative introductions of antimalarials to reduce the selective pressure on the parasite, has also been considered [20]. This raises several questions. Is the risk of drug resistance re-emergence too high? For how long should these cycles last, long enough so that resistance re-emergence is observed? Should these drugs be recycled on a regular basis? [20]. It has been demonstrated that *plasmepsin 2-3* gene amplification in DHA/PPQ resistant parasites is associated with *pfmdr1* gene single copies, so these resistant parasites are sensitive to mefloquine [4,83]. In contrast, ASMQ-resistant parasites with *pfmdr1* gene amplification are sensitive to piperaquine [3,4,24,83]. Based on the amplification of *pfmdr1* gene copy numbers of ACT-resistant parasites, the alternating use of ASMQ and DHA/PPQ is under consideration.

## Cross-resistance extension with other antimalarials

Artemisinin resistance is a major threat to global public health, and there is an urgent need to accelerate the elimination of *P. falciparum* in the greater Mekong subregion, where standard courses of ACTs are failing [77]. That is why malaria elimination requires, among other things, new, highly effective medicines. The global portfolio of antimalarial medicines contains 33 new medicines composed of various chemical entities with many mechanisms of action that have been evaluated from preclinical research to regulatory review [47]. However, new antimalarial drugs studies are highly challenged by the risk of cross-resistance with artemisinins.

How could extended artemisinin pressure affect the response of artemisinin-resistant *P. falciparum* to other antimalarial drugs in the field? It has recently been demonstrated *in vitro* that prolonged exposure to artemisinin induced a novel multidrug-tolerant phenotype in previously artemisinin-resistant parasites. After 5 years of artemisinin pressure, the resistant strain F32-ART5 was able to stop multiplying by entering into a quiescent state following treatment with artemisinin [85] as well as with other antimalarial drugs alone such as quinolines (amodiaquine, mefloquine, chloroquine, quinine) or pyrimethamine. Surprisingly, this pluri-resistance was not associated with any of the known genes involved in resistance to these drugs, except *pfk13* [44]. Only atovaquone escaped this multi-tolerance by remaining effective in the F32-ART5 parasite line [44]. This could be explained by its mode of action, which is based on the inhibition of mitochondrial electron transfer, which is actively maintained in quiescent parasites [15,57]. Unfortunately, resistance to atovaquone is easily and quickly selected in the field, independent of artemisinin use [31].

From a pharmacological point of view, compounds with similar chemical structures and modes of action (namely, ozonides and trioxaquines) raise concerns about cross-resistance. In fact, a recent laboratory study showed that artemisinin-resistant strains as well as resistant Cambodian isolates presented cross-resistance with trioxaquines (endoperoxide-based hybrid antimalarial molecules). Moreover, trioxaquine drug pressure selected *in vitro* a new lineage that was resistant to both trioxaquines and artemisinins, in a manner supported by *pfk13* polymorphism [56].

Moreover, among the most promising medicines of the antimalarial portfolio are two ozonide compounds OZ439 and OZ277, respectively known as artefenomel and arterolane, both of which contain an endoperoxide bridge, a chemical function also found in artemisinins. Although OZ439 has shown a good safety profile in a clinical trial and rapid parasitemia clearance in *P. falciparum* (and in *P. vivax*) [61], it was recently reported that this is not the case for artemisinin-resistant strains *in vitro*. In fact, resistant parasites can exhibit reduced *in vitro* sensitivity to the ozonide antimalarials, depending on the exposure time [89]. Moreover, it has been shown that ozonide OZ277 demonstrates significantly limited *in vitro* activity against artemisinin-resistant parasites, while OZ439 seems effective against most *pfk13*-mutated, artemisinin-resistant parasite lines, except for those harbouring the mutation I543T [72].

Therefore, all these data raise concerns about the risks of parasite cross-resistance between artemisinins and other endoperoxide-based antimalarials, including ozonides. Field-based resistance monitoring is indispensable to detect any cross-resistance in artemisinin-resistance areas. Furthermore, these results highlight the importance of investigating the ability of newly developed antimalarial drugs to select for resistance based on a quiescence mechanism.

## Risk of worldwide spread of artemisinin resistance

The greater Mekong subregion is the epicentre of the emergence of *P. falciparum* resistance, with the potential for it to spread to other malaria-endemic continents [46]. This regional specificity could be explained by many factors, such as host immunity levels leading to the regular use of antiplasmodial drugs, genetic parasite factors due to *Plasmodium* origins, poor access to drugs, and the continued use of monotherapy. Moreover, low quality and counterfeit antimalarial drugs are widespread. Recent estimates from Southeast Asia suggest that up to 50% of the artesunate sold is fake. This situation is expected to worsen in other endemic malaria countries with the implementation of ACTs, which are more expensive [30]. ACTs sold without any quality control are ubiquitous in Sub-Saharan Africa, both in the private and the public sectors. Kenya is a telling example since fake ACTs represent 20% and 5% of the antimalarial treatments sold by the country’s private sector and public sector, respectively [10]. In the seven African countries audited under the ACTwatch project (Benin, Democratic Republic of Congo, Kenya, Nigeria, Tanzania, Uganda and Zambia), non-quality-assured ACTs accounted for 32% to 89% of the total ACTs used, and, surprisingly, non-quality-assured ACTs were more expensive than the quality-assured drugs [52].

The consumption of poor-quality antimalarials in Sub-Saharan Africa has also affected children under five years of age, with an estimated 122,350 avoidable deaths in 2013 [66]. On the other hand, the continued use of monotherapy is widely considered to be one of the main factors contributing to the development and spread of resistance. Until December 2014, eight countries, mainly in Africa (Angola, Cape Verde, Colombia, Equatorial Guinea, Gambia, Sao Tome and Principe, Somalia, and Swaziland) still offered artemisinins in monotherapy as part of their health policy, despite their ban by the WHO [82].

The grave concern of artemisinin resistance spreading from Asia is further aggravated by the history of antiplasmodial drug resistance, such as the emergence of chloroquine resistance and its spread in the 1970s [46], and by the recent report of the ability of artemisinin-resistant parasites originating from Asia to infect and be transmitted by a wide range of *Anopheles* species, including the main African malaria vector, *Anopheles gambiae* [36].

From a molecular point of view, the independent emergence of artemisinin resistance outside Asia is dependent on the role of the genetic background of the parasite. Resistance to artemisinins is heritable and therefore has a clear genetic basis [5,62]. Genome modification studies have shown that the impact of various *pfk13* mutations on parasitic clearance and the survival rates of ring stage parasites is dependent on the genetic background of the parasites [73]. Artemisinin resistance appears to have been selected from a population of predisposed parasites with polymorphisms of the *fd* (ferredoxin), *arps10* (apicoplast ribosomal protein S10), *mdr2* (multidrug resistance protein 2) and *crt* (chloroquine resistance transporter) genes [45]. Another study suggested that mutations in a number of DNA repair genes such as *mlh1*, *pms1* and *exo1,* are overexpressed in artemisinin-resistant parasites [40]. Together, these data indicate that the risk of emergence of new mutations causing resistance to artemisinins is promoted by specific genetic factors in a parasite population [14,45].

However, no correlation between drug resistance emergence and increased mutation rates of the parasite’s genome was found in Southeast Asia, invalidating the hypothesis of a "hypermutator" parasite [11]. In contrast, evidence of a "mild mutator" phenotype has been shown in two artemisinin-resistant Cambodian isolates [40].

Spatiotemporal analysis of many isolates collected in Cambodia over the past decade has shown a gradual increase in the frequency of mutant K13 parasites in resistance-affected provinces [4,6]. However, to date, the KARMA (K13 Artemisinin Resistance Multicenter Assessment) study, analysing more than 14,000 parasite samples from 59 countries in which malaria is endemic, determined, on the basis of *pfk13* monitoring, that artemisinin resistance is confined to Southeast Asia and has not yet spread and/or emerged in other endemic malaria areas, i.e., Sub-Saharan Africa, South America and Oceania [43]. Only one suspected case of artemisinin resistance (associated with a non-synonymous single nucleotide polymorphism (M579I) K13 mutation) has been reported in Africa in a man who returned to China after having worked for 20 months in Guinea [41]. So far, there has been no confirmed resistance to ACTs or delayed parasite clearance during routine therapeutic efficacy studies conducted in Africa [54,70]. One study suggested that a threshold of 5% of cases on day 3 with parasite positivity is more suited to artemisinin resistance monitoring in Sub-Saharan Africa due to the higher levels of acquired immunity against *Plasmodium* in African populations, which contributes to faster parasite clearance [87]. Furthermore, for ACT treatments, several factors could also influence parasite clearance time values such as initial parasite biomass and partner drug efficacy, as well as, for artemisinin-sensitive areas, patient age, health status (i.e., fever with a possible relation with schizont rupture) and artemisinin dose [88]. All these data could be integrated in artemisinin-resistance emergence monitoring. Indeed, recent studies showed that low levels of immunity are correlated with a high prevalence of *pfk13* mutations across the greater Mekong subregion. This may be due to a drug pressure-independent mechanism that could be linked to the fitness of resistant or wild-type parasitic populations, depending on levels of immunity and transmission [7,8]. Moreover, the emergence of *pfk13* mutations in Asia was preceded by a gradual decrease in both transmission and immunity in the previous 6 years [8]. In South America and Oceania, the absence of artemisinin resistance may also be explained by the more recent introduction of ACTs.

To date, Africa is an area with a high malaria transmission rate and high naturally acquired immunity. However, with the support of the WHO programme (Global Technical Strategy for Malaria 2016–2030), the progress made by countries in malaria elimination and the decrease in malaria incidence are expected to be associated with a decrease in immunity levels, which may provide conditions conducive to the emergence of artemisinin resistance. If resistance to artemisinin and ACTs were to emerge in Africa, where 90% of deaths occur [79], this could have a devastating impact on malaria-related morbidity and mortality.

Across Africa, it is estimated that if ACT resistance were similar to the highest levels of artemisinin and partner drug resistance currently observed in Cambodia, there would be 78 million additional clinical malaria cases between 2016 and 2020 [69]. Medical costs for the treatment of clinical failures and for the management of severe malaria will also increase. The spread and/or emergence of artemisinin and ACTs resistance will also lead to a loss of  productivity resulting from excess morbidity and mortality [42].

Based on these results, it is important to pay considerably more attention to control of the emergence of artemisinin resistance and to invest greater resources than those currently being made available.

## Conclusion

The emergence of resistant parasites to both artemisinins and partner drugs, as well as the lack of the short-term availability of effective alternative antimalarial drugs, are of great concern in the fight against malaria. Furthermore, the development of multi-tolerance by *P. falciparum* in the field, which has also been demonstrated *in vitro* after several years of drug pressure with artemisinin alone, should be a major concern for government and international authorities. Although there is no current evidence that artemisinin resistance has emerged outside Asia, this finding reinforces the need for routine monitoring and surveillance of the therapeutic efficacy and safety of artemisinins and ACTs, as recommended by the WHO, for effective case management and early detection of resistance. This artemisinin resistance monitoring should include the proportion of patients with early treatment failure, late clinical failure or any inadequate clinical response, the differentiation between recrudescence and new infection, the polymorphism of the molecular marker K13, and the *in vitro* susceptibility of *P. falciparum* isolates to artemisinins by relevant assays.

## Conflicts of Interest

The authors declare that they have no conflicts of interest in relation to this article.
